# Two Types of Motor Strategy for Accurate Dart Throwing

**DOI:** 10.1371/journal.pone.0088536

**Published:** 2014-02-12

**Authors:** Daiki Nasu, Tomoyuki Matsuo, Koji Kadota

**Affiliations:** Graduate School of Medicine, Osaka University, Toyonaka, Osaka, Japan; VU University Amsterdam, Netherlands

## Abstract

This study investigated whether expert dart players utilize hand trajectory patterns that can compensate for the inherent variability in their release timing. In this study, we compared the timing error and hand trajectory patterns of expert players with those of novices. Eight experts and eight novices each made 60 dart throws, aiming at the bull’s-eye. The movements of the dart and index finger were captured using seven 480-Hz cameras. The data were interpolated using a cubic spline function and analyzed by the millisecond. The estimated vertical errors on the dartboard were calculated as a time-series by using the state variables of the index finger (position, velocity, and direction of motion). This time-series error represents the hand trajectory pattern. Two variables assessing the performance outcome in the vertical plane and two variables related to the timing control were quantified on the basis of the time-series error. The results revealed two typical types of motor strategies in the expert group. The timing error of some experts was similar to that of novices; however, these experts had a longer window of time in which to release an accurately thrown dart. These subjects selected hand trajectory patterns that could compensate for the timing error. Other experts did not select the complementary hand trajectories, but greatly reduced their error in release timing.

## Introduction

Throwing accurately has been an important skill in many situations, from the Stone Age to present-day sports. For Stone Age hunters, the ability to throw accurately might have been a requisite skill to hunt prey from long distances. For baseball, basketball, and dart players, accurate throwing or shooting strongly influences the outcome of the game. In the current study, we investigated dart throwing as a typical example of an accurate throw. What are the motor mechanisms that characterize an accurate throw?

The place where a thrown dart will hit is physically determined by a combination of release parameters, including the position, velocity, and direction of motion at the moment of release, assuming that both the rotation and air resistance are negligible. These parameters depend on the hand trajectory of the throwing arm and the release timing. In order to reduce the variability across repeated throws, it is important that throwers reduce the variability in both the hand trajectory and release timing.

Several studies have shown that precise control of the release timing is the most important factor for an accurate throw. Hore et al. [1], [2] reported that the accuracy in overarm ball throwing was most closely related not to the hand trajectory variability, but to the timing precision, which was measured with respect to the moment when the hand had a vertical position in space. Hore et al. [3] stated that baseball pitchers require a timing precision of 1–2 ms to hit the strike zone consistently. For dart throwing, good throwers theoretically need a timing precision of 1.8 ms or less in order to hit a bull’s-eye with a diameter of 4.4 cm [4]. Simulation studies have also suggested that throwers need to release within a window as short as 1 ms to hit a target 20 cm in diameter, positioned at a distance farther than 6 m [5], [6].

On the other hand, Newell and Corcos [7] said that “variability is inherent within and between all biological systems.” It has been considered that trial-to-trial variability has been observed in human behavior, because noise exists at all levels of the nervous system (e.g., synaptic, neural, and muscular noise) [8]. Based on this supposition, it would be surprising if the central nervous system could control the variation in the finger release timing for a certain event within 1–2 ms, from throw to throw. Indeed, behavioral studies have reported greater variability in the release timing than the theoretically permissible range (e.g., 9 ms in a virtual throwing task [9], 7–10 ms for ball throwing by recreational players [1], [2], [10], and 27 ms for ball throwing by unskilled subjects [11]).

Two previous studies suggested that the timing variability can be compensated for by modifying the hand trajectory. Müller and Loosch [12] demonstrated that throwers developed movement patterns that maximized the range of release for accurate outcomes in a single-joint virtual throwing task. This means that the throwers learned a movement pattern that could reduce the sensitivity to timing errors, which led to success without precise timing control. Cohen and Sternad [9] further developed Müller’s study and provided evidence for their claim. They reported that in a virtual throwing task, throwers learned more accurate timing and improved their performance, but their improvement reached a plateau. With long-term, additional practice, the throwers in the study optimized their hand trajectory patterns to compensate for the inherent limitations in the release timing. However, it seems premature to accept their results for the general case, because the virtual throwing tasks in the cited studies were more constrained and specific than a real throwing task.

Smeets et al. [4] presented experimental data and simulations for real dart throwing and found results contradictory to those reported in Müller’s [12] and Cohen’s studies [9]. With practice, the subjects reduced their hand trajectory radii, and their releases changed, approaching the zeniths of their hand trajectories. These changes did not decrease the sensitivity to errors in the release timing; instead, the changes increased it. Only four subjects participated in Smeets’s study. Their experience throwing darts ranged from as much as “a few times a month” to as little as “a few times ever,” according to their self-reports. The subjects in the cited study might have been at the stage before learning the hand trajectory patterns that could reduce the sensitivity to timing errors.

Because the timing variability cannot be completely eliminated, as mentioned above, expert throwers are likely to select the most beneficial hand trajectory that compensates for the timing variability. The current study investigated whether expert dart players utilize a hand trajectory that can compensate for the inherent variability in their release timing. We tested several hypotheses by comparing expert players with novices. In several throwing tasks, it was reported that the movement variability decreased with practice, for example, in the joint kinematics [13], [14], release parameters [15]–[17], and release timing [9]. Therefore, we predicted that expert players have less variability in their release timing than that of novices. However, it is unlikely that throwers can control the release timing variability within 1 ms. The following hypotheses were examined in this study: 1) expert players show smaller variations in timing than novices, but not on the order of 1 ms, and 2) expert players have learned, through practice, the hand trajectory patterns that are more complementary to their release timing variability.

Because the horizontal hand trajectory in dart throwing is in near perfect alignment with the intended target, timing imprecision hardly influences the horizontal outcome [4], [12]. Therefore, we only focused on the movement and outcome in the vertical plane.

## Materials and Methods

### Subjects

An experiment was performed with 16 males. Eight right-handed dart players participated in the experiment as an expert group, with ages ranging from 25 to 49 years old. They were competitive soft-darts players, and their experience playing darts ranged from 2 to 6 years. The other eight right-handed adults (students and faculty) participated as a novice group. Their ages ranged from 19 to 37 years old. Each of the novices had only played darts a few times before. All of the subjects were provided written informed consent prior to the experiment. The study was approved by the ethics committee at the Graduate School of Medicine, Osaka University, and all procedures were in accordance with the Declaration of Helsinki.

### Task and Apparatus

Each subject performed all of their dart throws at a dartboard positioned according to the general soft-dart’s rules; the height of the center of the dartboard (the bull’s-eye) was 1.73 m above the floor, and the horizontal distance from the throwing line to the front of the board was 2.44 m. Subjects were free to choose their posture when throwing and were asked to always aim at the center of the bull’s-eye, which had a diameter of 4.4 cm. After 10–20 practice throws, each subject performed 20 sets, and each set consisted of three throws, giving a total of 60 throws.

**Figure 1:**
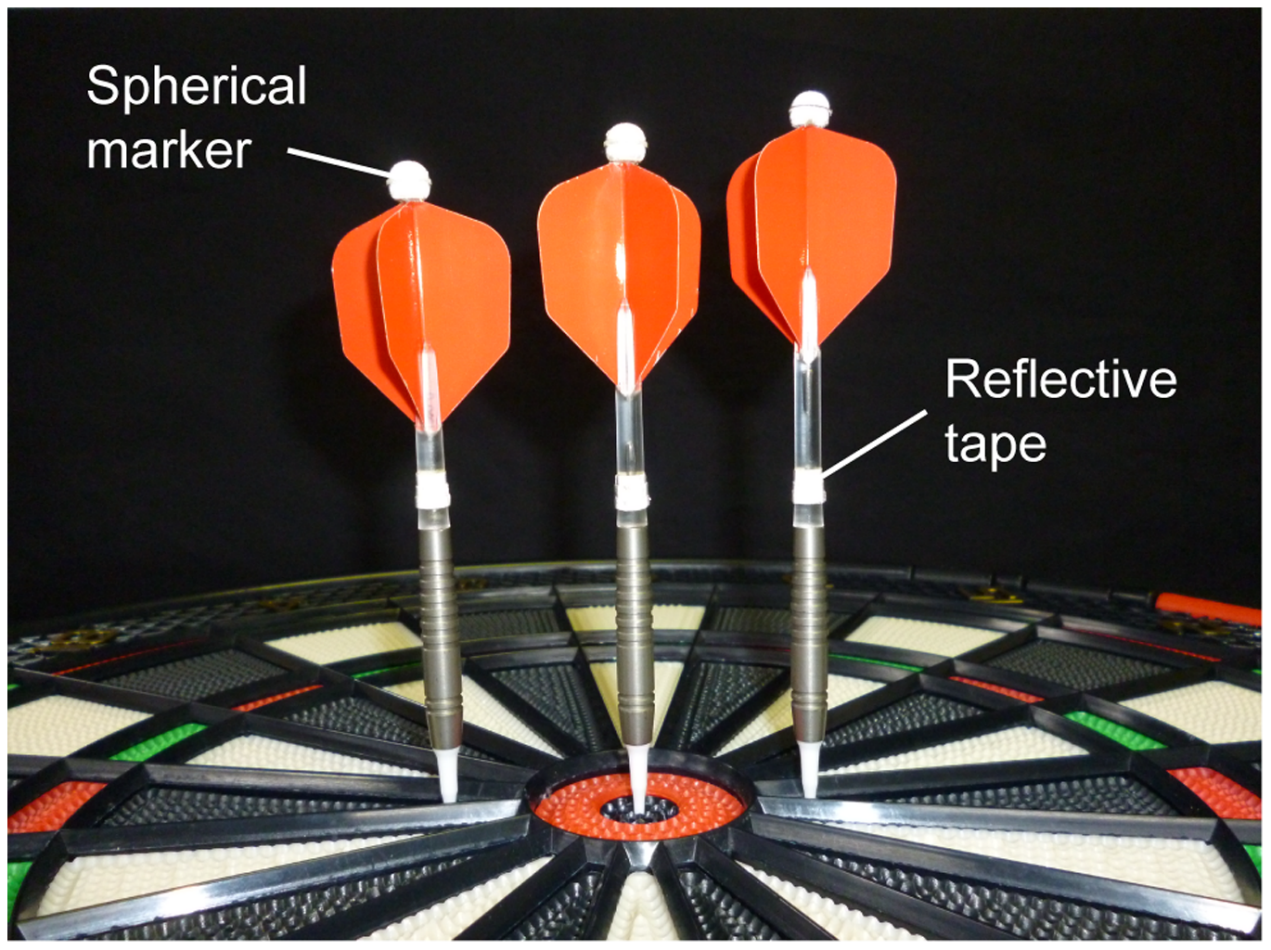
Darts with two landmarks. Starting from left, short, medium, and long darts are shown. Each subject chose a preferred size.

The subjects were asked to choose a preferred dart among the three different variations provided (long: length = 14.8 cm, weight = 18.5 g; medium: 14.0 cm, 18.2 g; short: 13.2 cm, 18.0 g). To capture the dart movement, we attached a spherical marker (r = 3.5 mm) to the rear of the dart, and wound reflective tape around the middle of the dart (Figure 1). Although the provided darts were slightly different from the darts that are normally used by experts, the experts said that they had been able to adapt to them through several practice throws. Five spherical markers were also attached to the tip of each subject’s index finger (r = 3.5 mm) and the joint centers of the metacarpophalangeal (MP), wrist, elbow, and shoulder of the throwing arm (r = 10 mm). The marker movements were captured using seven 480-Hz infrared cameras (Oqus300, Qualisys, Inc.). The mean ± standard deviation (SD) reconstruction error of the calibration frame was 0.45±0.05 mm.

To identify the actual location hit by each of the darts, the dartboard was recorded using a 30-Hz video camera (GZ-MG40, Victor, Inc.) that was positioned behind the subjects during the experiment. The location where the dart hit was manually digitized and calculated using two-dimensional direct linear transformation. The mean ± SD reconstruction error was 0.88±0.19 mm. These data were only used to compare with the calculated location on the board (see the section, “Time-Series Calculation of Vertical Errors”).

### Data Processing

The captured data were filtered using a Singular Spectral Analysis technique [18], in which the window length was a quarter of the data size, and two to six principal components were used. After the data smoothing, we interpolated the data at 1000 Hz using a cubic spline function to determine the timing variability with a precision of 1 ms. The movement of the center of gravity (CG) of the dart was calculated by the movements of the two landmarks on the dart, and the relative position of the dart’s CG, measured directly beforehand. The release time was defined as the moment when the velocity of the dart’s CG relative to the index finger exceeded a pre-determined threshold. Because the relative velocity for some subjects fluctuated before the release, a different threshold was selected for each subject (0.22–0.46 m/s). We conducted data processing using MATLAB (Math Works, Inc., Natick, MA).

### Time-Series Calculation of Vertical Errors

**Figure 2:**
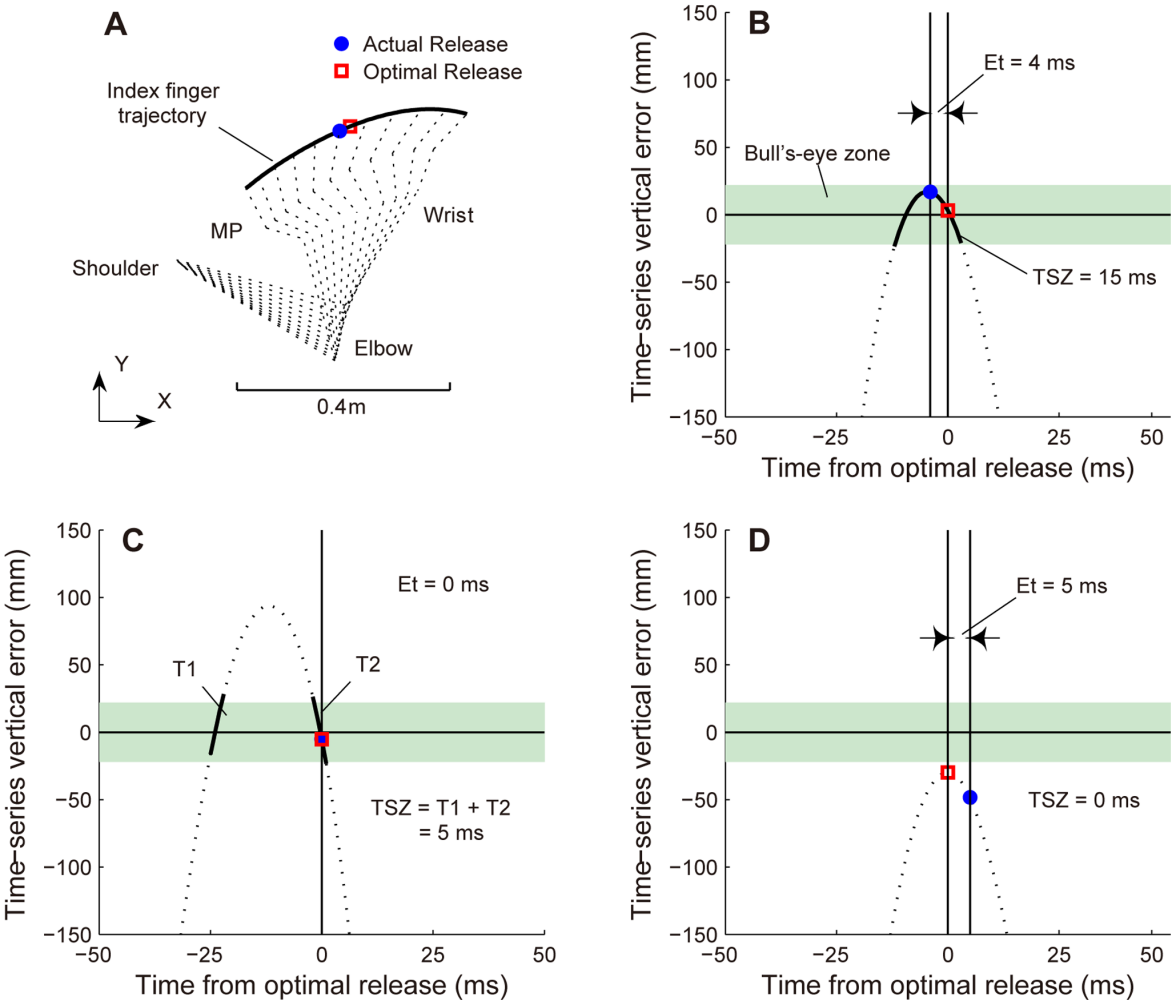
Example of time-series vertical error curves and definitions of relevant variables. A: Shown is an example of the index finger trajectory, from the side-view during a period extending 50 ms before and after the actual release. The dashed line indicates a stick figure of the throwing arm at 10-ms intervals. The coordinate data of the MP, wrist, elbow, and shoulder were only used to draw the stick figure. B–D: A curved line represents the time-series vertical error, which was calculated from the index finger movement (position, velocity, and direction of motion). The horizontal solid line indicates where the vertical error is zero. The horizontal green shade indicates the success zone for the required target. Timing error (Et) was defined as the absolute difference in time between the actual and optimal releases. The time in success zone (TSZ) was defined as the amount of time that the curve was in the success zone, and it is shown with bold black lines. When the curve crosses the zone twice, TSZ is quantified as the sum of two values (C). When the curve does not enter the zone, TSZ is zero; we called this trajectory a “non-hit trajectory” (D).

We needed to calculate the vertical error on the board when the thrower released at every point in the hand trajectory. Therefore, we calculated the vertical error using the index finger movement. Using the index finger allowed us to estimate the vertical error as a time-series error, including the time after the actual release (Figure 2). We assumed that the dart moved exactly with the index finger, and that the dart followed the parabolic trajectory of a point mass after release, neglecting air resistance and any rotation. At time *t*, the equation for the vertical error (*Ey*) on the board was written as follows:
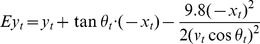
(1)where *x_t_* and *y_t_* are positions relative to the center of the bull’s-eye; *v_t_* is the velocity; and *θ_t_* is the direction of motion of the index finger in the vertical plane, at time *t*. This calculation was based on the analyses in the studies of Smeets et al. [4], and Cohen and Sternad [9].

Because a few throws resulted in an unacceptable difference between *Ey_t_* and the error obtained from the manually digitized data (>4 cm), the outlying throws were eliminated from the analysis. The mean ± SD of the number of eliminated trial throws for each subject was 3.6±3.4. The calculated locations correlated well with the digitized locations (the correlation coefficient was 0.86±0.14). The standard deviation of the difference between the calculated and digitized locations was 18.5±2.8 mm.

### Dependent Variables

An example of the index finger trajectory, with a stick figure of the throwing arm, is shown in Figure 2A. The time-series vertical error, shown in Figure 2B, was calculated from the index finger trajectory. Other typical examples of the time-series vertical error are shown in Figure 2C and 2D. We calculated the following variables related to the timing variability and hand trajectory pattern, based on the time-series vertical error.

#### Performance outcome

We calculated two variables defined as the performance error and success rate to assess the throwing accuracy in the vertical plane. Because we focused on the vertical plane, a successful trial in the current study was defined by the case when the vertical outcome matched the bull’s-eye height, independent of the horizontal outcome. Therefore, the performance error was defined as the absolute value of the vertical error, *Ey,* in equation (1), at the actual time of release. The success rate was defined as the ratio of the number of throws with a performance error lower than 22 mm to the total number of throws for each subject.

Generally, the skill level of the players in a dart game can be judged by the number of hits in closest proximity to the target center, including the horizontal plane. Our two vertical variables, the performance error and success rate, strongly correlated with the bull’s-eye hit rate (performance error: *r* = –0.90, success rate: *r* = 0.95). Therefore, our two variables are reasonable metrics for judging and comparing the performances of the groups.

#### Timing precision

We calculated the timing error (Et) to evaluate the accuracy of the release timing. Several previous studies investigated the release timing precision. In these studies, the release timing was synchronized with a kinematic landmark (e.g., when the hand was vertical in space [10], [19]). Then, the timing variability was calculated using the standard deviation. However, Smeets et al. [4] found that this method was not adequate to describe the timing accuracy, because the kinematic landmark used as a reference was also variable. Cohen and Sternad [9] employed another method to quantify the timing accuracy. They determined the “timing error” as the absolute difference between the actual and optimal releases, the release resulting in the minimum error during the corresponding throw. This method can evaluate whether throwers release the projectile at the optimal moment, within the hand trajectory for each throw. In the current study, the timing error was calculated based on the analysis in Cohen’s study. When the time-series error crossed the “0” line twice, the moment that was closer in time to the actual release was selected as the optimal release (Figure 2). Then, Et was calculated as the absolute difference between the optimal and actual releases. The value was averaged across all throws for each subject.

Because Et is not a measure of dispersion, it is difficult to compare the timing precision with that of the previous studies. Thus, we also calculated the timing variability as the standard deviation of the release timing with respect to the zenith of the hand trajectory [4] in order to compare our result with those of other studies.

#### Time in success zone

We wanted to clarify whether a subject had the hand trajectory patterns that could compensate for the timing variability. To accomplish this, we quantified the time in success zone (TSZ) as the duration for which the curve of the time-series vertical error was in the region in which a release would result in success (the success zone). The curve of the time-series vertical error represents the hand trajectory patterns, where a longer TSZ means that the particular hand trajectory pattern has a longer release time window that can lead to success. When the curve crosses the success zone twice, TSZ is quantified as the sum of the two values (Figure 2C). When the curve does not enter the zone, TSZ is zero; we called this trajectory a “non-hit trajectory” (Figure 2D).

TSZ was averaged across successful throws, for each subject. If failed throws, resulting from “non-hit trajectories,” were included in this variable, the average TSZ would be skewed because in these cases it was assigned a value of zero. Therefore, averaging all the throws of each subject does not truly reveal the regular hand trajectory patterns. The number of successful throws for the expert subjects ranged from 25 to 57, whereas the number for novices ranged from 8 to 24.

#### Variability in curve of time-series vertical error

The curves of the time-series vertical error represent the patterns of the hand trajectory, including the position, velocity, and direction of motion. Therefore, the variability in these curves for each subject represents the variability in the hand trajectory patterns. In order to estimate this, we calculated the standard deviations of the peaks of the time-series error curves because these suitably indicate the characteristics of these curves (Figure 2B–D). We calculated the standard deviations of the peak values and peak times, which synchronized with the time of optimal release, for each subject.

### Statistics

Wilcoxon rank-sum tests were used to assess the differences among the six variables, between the expert group and the novice group. The statistical significance was set at *p*<0.05.

## Results

### Performance Outcome

**Figure 3:**
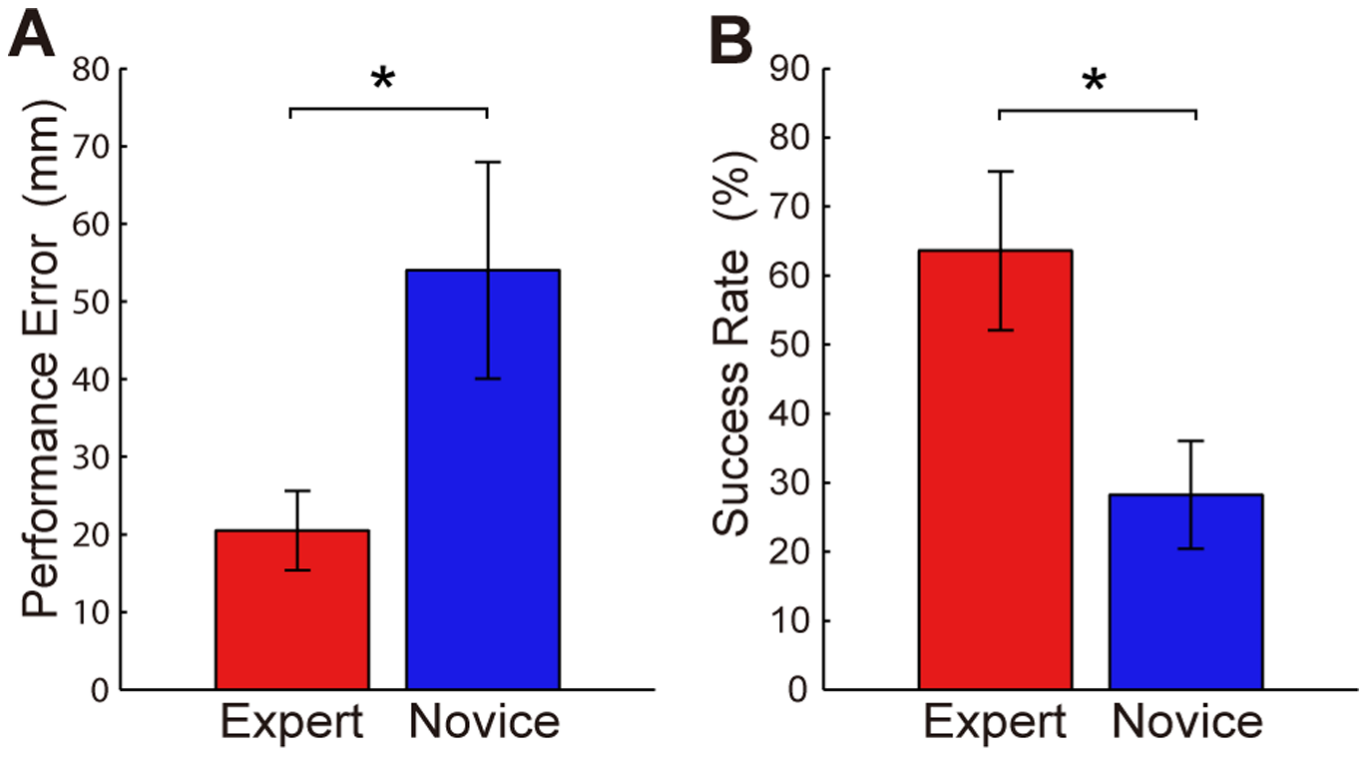
Mean performance error and success rate. The performance error (A) and success rate (B) were averaged per group and are indicated with bars. The error bars denote standard deviations between subjects. *: *p*<0.05.

The mean value of the performance error was significantly smaller for the expert group than for the novice group (Figure 3A: *W* = 36, *p*<0.05). The mean value of the success rate was significantly higher for the expert group than for the novice group (Figure 3B: *W* = 100, *p*<0.05). The best performer among all the subjects was Expert 1 (performance error = 13.5 mm, success rate = 77.0%) with respect to the performance error, and Expert 2 (performance error = 14.4 mm, success rate = 79.2%) relative to the success rate. These subjects will be used as successful examples in a later section. According to both measures, the worst expert performed better than the best novice. This confirmed that long-term practice improves throwing accuracy.

### Typical Patterns of Time-Series Vertical Error

**Figure 4:**
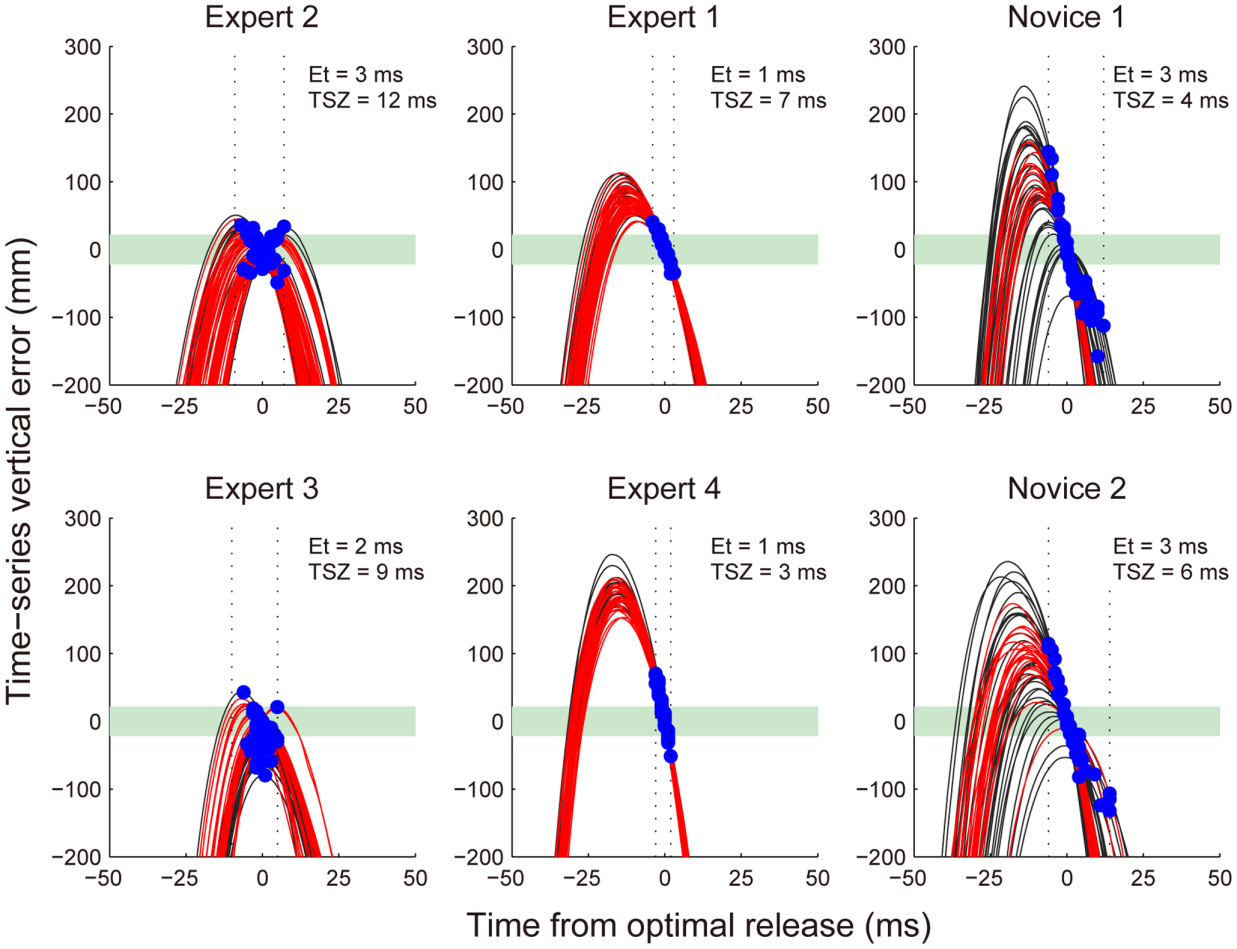
Examples of time-series vertical error. The time-series error curves for four experts and two novices. The time was synchronized with the optimal release. Red line: successful throw. Blue dots: actual release moment. Green shading: success zone. Vertical dotted lines: range of Et.

We found two typical types of strategies in the expert group and another type in the novice group. Three typical exemplary curves of the time-series vertical error for all throws by each subject are shown in Figure 4. In the current study, we calculated the vertical error as a time-series, using the index finger movement. Thus, the curves of this time-series error represent the hand trajectory patterns, including the position, velocity, and direction of motion. One of the typical patterns, illustrated in the left column of Figure 4, shows relatively consistent throws and overlaps with the success zone for a significant amount of time (i.e., the patterns demonstrated by these subjects had longer TSZs). This means that these subjects had a longer time release window that could produce an accurate outcome. However, the range of values for Et for these subjects was similar to the range of values for the novices. That is, they had hand trajectory patterns that could compensate for the timing error of the release. The second set of exemplary curves, illustrated in the middle column of Figure 4, shows high consistency. The duration of TSZ for these subjects was similar that of TSZ for the novices, but their release timings were concentrated around the optimal release. Thus, Et was small. These subjects did not select a complementary hand pattern, but had extremely reduced timing error, resulting in accurate outcomes. The third type of curve, illustrated in the right column of Figure 4, shows greater variability, lower TSZ values, and a wider range of values for Et. This pattern was only demonstrated by the novices. Novices could not achieve consistently accurate outcomes because they had neither complementary hand patterns nor small error in their release timings.

### Comparisons between Expert and Novice Groups

**Figure 5:**
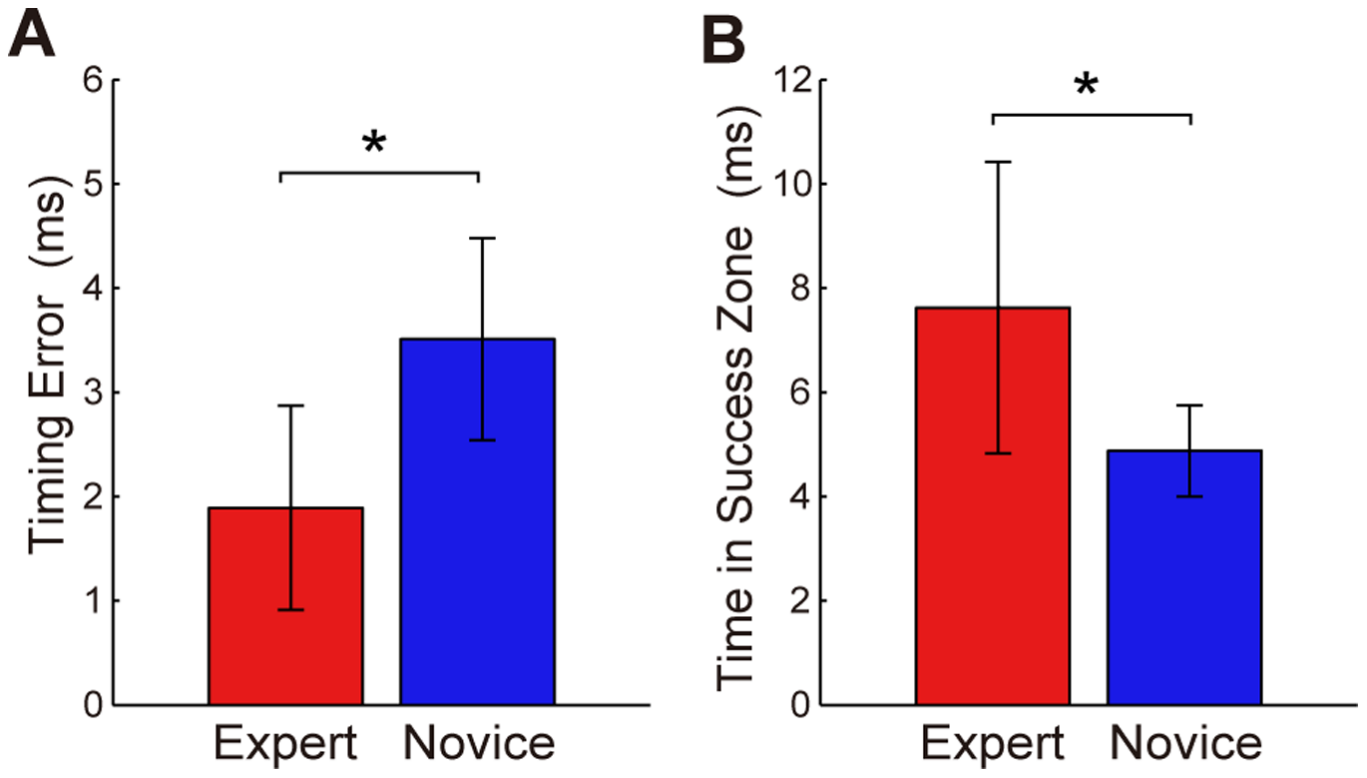
Mean timing error and time in success zone. The timing error (A) and time in success zone (B) were averaged per group and are indicated with bars. The error bars denote standard deviations between subjects. *: *p*<0.05.

**Figure 6:**
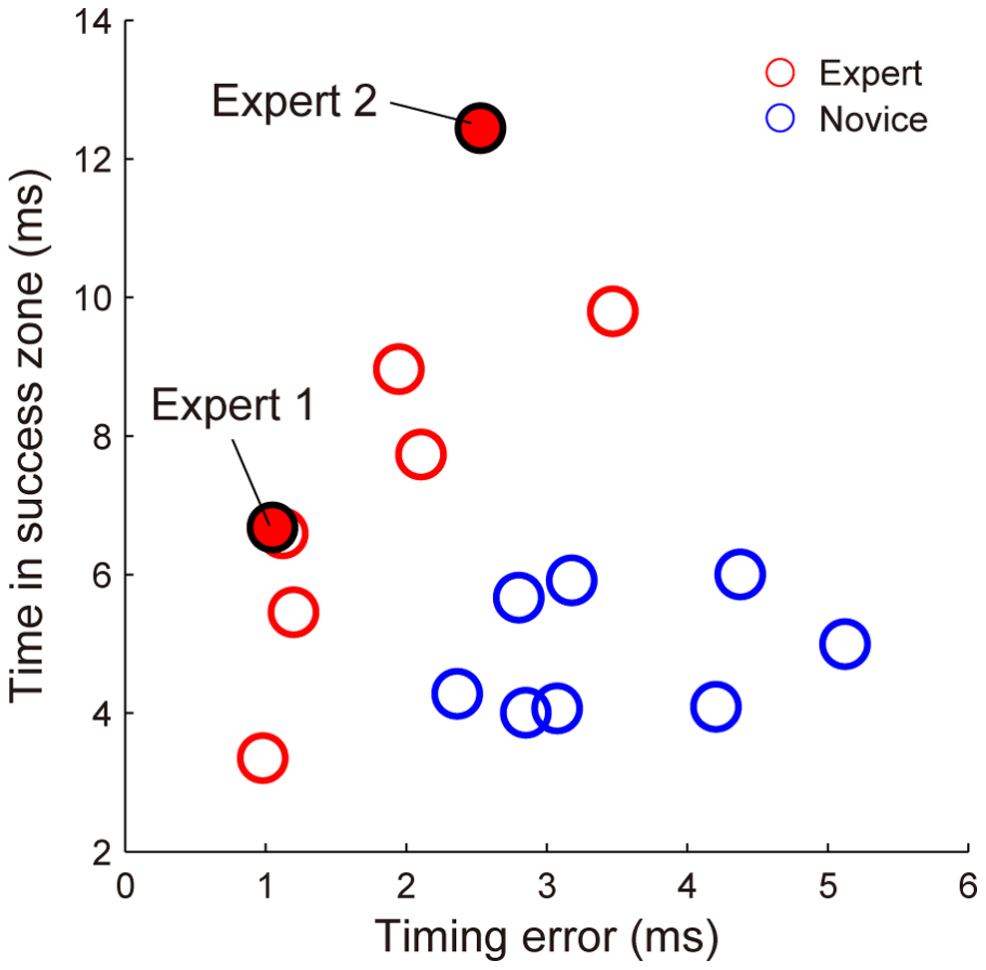
Characteristics of timing error and time in success zone for each subject.

The mean value of the timing error (Et) was significantly smaller for the expert group than for the novice group (Figure 5A: *W* = 42, *p*<0.05). That is, the experts released the darts closer to the point that led to the minimum error on each throw. However, some experts demonstrated throws with values of Et similar to those of the novice group (Experts 2 and 3 in Figure 4), and some experts demonstrated extremely small values of Et, on the order of 1 ms. Expert 4 had the smallest value, 0.98±1.13 ms (Figure 4), and four of the eight experts showed values of Et smaller than 1.2 ms (Figure 6).

The experts showing small values for Et had relatively short TSZs (Figure 6). Their TSZs did not differ from those in the novice group. On the other hand, the experts showing the same Et as the novice group had higher values for TSZ. The mean value for TSZ was significantly higher for the expert group than for the novice group (Figure 5B: *W* = 89, *p*<0.05) even though half of the experts demonstrated values similar to those of the novice group. This occurred because the other half of the experts had large TSZs. Expert 2 had the highest TSZ value (12.4 ms) among all the subjects. This means that Expert 2 had a release window that could result in accurately hitting the target that was as long as 12.4 ms.

Thus, we found two types of strategies in the expert group. Each of the two experts showing the best performances (Experts 1 and 2) selected one of these strategies.

The mean of the variability in the peak value of the time-series error curves was significantly smaller for the expert group than for the novice group (experts: 21.3±4.3 mm, novices: 65.0±13.0 mm; *W* = 36, *p*<0.05). The mean of the variability in the peak time with respect to the optimal release time was also significantly smaller for the expert group than for the novice group (experts: 2.1±1.2 ms, novices: 4.2±0.7 mm; *W* = 39, *p*<0.05). Then, the hand trajectory patterns for the experts were more stable, both spatially and temporally, than those for the novices.

### Performance Error and Relationship between Et and TSZ

**Figure 7:**
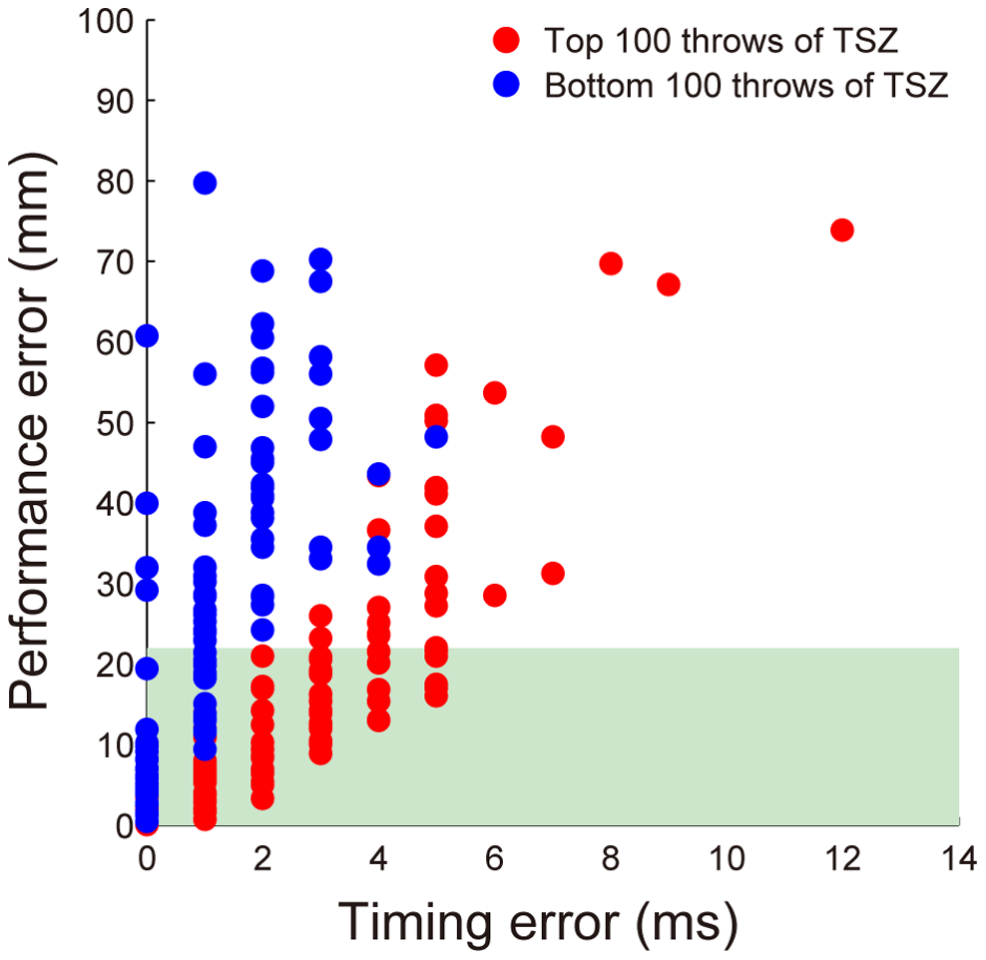
Sensitivity of timing error to performance error: Difference between lengths of time in success zone. Relationship between timing error and performance error of all throws from all experts. The red dots indicate the top 100 throws, which have longer TSZs, and the blue dots indicate the bottom 100 throws.

In order to confirm that larger TSZs actually compensated for the Et, we plotted the relationship between Et and the performance error for all of the throws from the experts, with discriminating between the longest TSZ and the shortest TSZ (Figure 7). Red dots indicate the top 100 throws in TSZ (mean ± SD: 13.3±2.2 ms), and blue dots indicate the bottom 100 throws in TSZ (2.7±1.6 ms). The number of the total successful throws for the top 100 throws in TSZ (76 throws) was significantly greater than that for the bottom 100 throws in TSZ (44 throws) [two-sample proportion test, *x* ^2^ = 21.3; *p*<0.05]. Thus, throws with the highest values of TSZ had a low sensitivity to Et. On the other hand, the performance error for the throws with the shortest TSZ increased drastically as Et increased.

### Relationship between Et and Timing Variability

**Figure 8:**
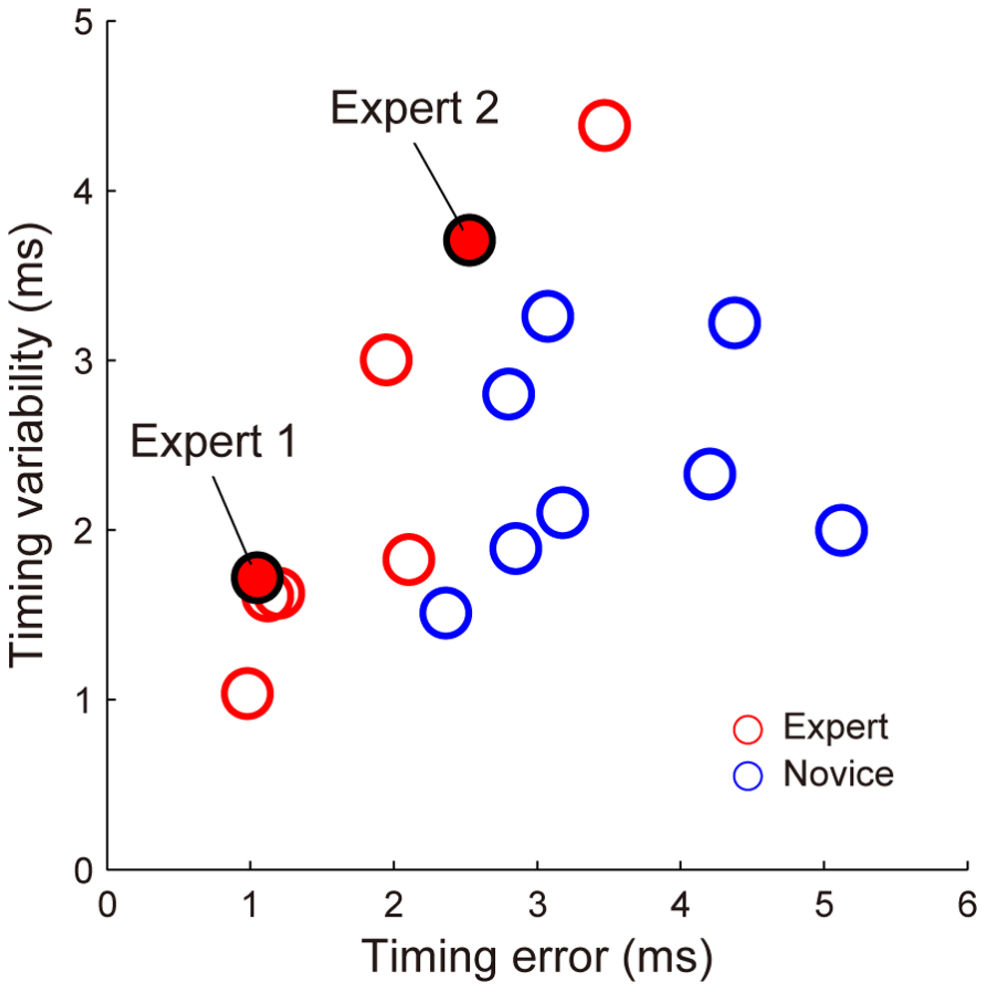
Relationship between timing error and timing variability. Each circle indicates the data for one subject. The timing error was the absolute difference between the actual and optimal releases. The timing variability was the standard deviation of the actual release moment with respect to the zenith of the hand trajectory.

Another estimation for the timing precision was the timing variability. The mean values were not statistically different between the expert and novice groups (expert: 2.4±1.2 ms, novice: 2.4±0.6, *W* = 62, *p* = 0.57). There was a strong correlation between Et and the timing variability for the experts (*r* = 0.93), but there was no correlation for the novices (*r* = 0.19) (Figure 8). It was shown that some novices demonstrated small timing variability despite a large Et.

## Discussion

The aim of the current study was to investigate whether expert dart players have hand trajectory patterns that can compensate for the inherent variability in their release timing. We compared the timing precision and hand trajectory patterns between expert players and novices. The results demonstrated that the expert group had less error in their release timing and more complementary hand trajectory patterns than the novice group. These results supported our hypothesis. However, we found two typical types of throwing strategies in the expert group. In particular, the two throwers that performed the best (Experts 1 and 2) selected distinctly different strategies. The characteristics of these strategies are discussed below.

### Strategy for Complementary Hand Trajectory Pattern

One strategy involved hand trajectory patterns that could compensate for the timing variability to consistently produce accurate throws. Expert 2 used this strategy, and even though the timing errors he demonstrated were not different from the novices’ errors; he compensated for the timing error with the modified hand trajectory. This result is consistent with the results of Cohen and Sternad’s study [9], which demonstrated that skilled subjects optimized their trajectory to compensate for intrinsic limitations in timing variability. They mentioned that a key advantage of this strategy is that the trajectory can be planned in advance, and the thrower does not have to rely on feedback from the current throw. Dart throwing movements are too fast to make corrections based on proprioceptive information [20]–[22]. It can be speculated that the subjects using this strategy performed pre-planned hand trajectories, which could reduce the sensitivity to the release timing variability.

### Strategy for Reducing Variability in Release Timing

Another strategy involved reduced timing variability for improving throwing accuracy. Four experts, including Expert 1, demonstrated this strategy and reduced their timing error to as low as 1 ms. This strategy was used instead of a complementary hand trajectory pattern in order to achieve accurate throws.

Calvin [5] and Chowdhary and Challis [6] reported that the release window theoretically needs to be shorter than 1 ms to achieve a high level of accuracy. Hore and Watts [23] demonstrated that university baseball players demonstrated a series of successive throws with release timings as low as 1 ms. The results for our four experts that demonstrated the strategy of reducing Et seem to support the results of these previous studies [5], [6], [23]. However, because of differences in the definitions of timing accuracy, our timing error results cannot simply be compared with those of the previous studies, which estimated the timing variability as the standard deviations with respect to a kinematic landmark. Therefore, we also calculated the timing variability synchronized with the zenith of the hand trajectory. The timing variability of our four experts was 1.0–1.7 ms. This timing variability result was lower than the results of the study by Smeets et al. in dart throwing (3.4–7.7 ms), and their timing variability definition was the same as ours [4]. However, it was slightly larger than the results of the study by Hore and Watts (average 0.84 ms). This value was estimated from their result, the timing window for 95% of throws (SD×3.92) for a ball release with respect to vertical hand position [24]. Hore and Watts mentioned that precise timing control came from a mechanism involving reactive forces. The skilled throwers in their study achieved timing control with a variability of 1 ms by computing the finger force/stiffness, which was based on an estimation of the backforce exerted by the ball owing to hand acceleration. Because dart throwing differs from ball throwing in terms of the projectile weight, arm acceleration, and finger direction during arm acceleration and at the moment of release, the perceptible backforce at the finger-tip would be much smaller. However, it is probable that these are the same mechanisms. Cohen and Sternad reported that the timing error plateaued at 9 ms in their virtual throwing task [9], which was considerably larger than our result for Et. The large timing error in the study of Cohen and Sternad might be the result of not including a “backforce mechanism.” In their task, the release was determined based on the extension of the index finger.

### Measures of Timing Precision

The values of Et were significantly different between the expert and novice groups, but the timing variability values were not different. This was because the experts with larger Et values also had larger timing variability values, but some novices had smaller timing variability values despite larger Et values (Figure 8). That is, these novices demonstrated rather good spatial control of the release synchronized with the zenith of their hand trajectory. However, their hand trajectory patterns, including the velocity and direction of motion, were highly variable. The variability of hand trajectory patterns influence the moment of optimal release, then do Et.

The experts who reduced their timing variability showed very little variability in both Et and the timing variability (e.g., Expert 1 had an Et of 1.0 ms and a timing variability of 1.7 ms). Thus, these experts moved their hands stereotypically and showed closer coupling of the timing with the hand trajectory. On the other hand, the experts who used complementary hand trajectory patterns showed larger variability, both in Et and the timing variability (e.g., Expert 2 had an Et of 2.6 ms and a timing variability of 3.7 ms). These experts reduced the sensitivity to the timing variability.

In this manner, the timing precision value was influenced by the landmark that was used (the optimal release or the zenith of the hand trajectory). It is impossible to say which measure is better because this depends on the purpose of the research.

### Which Strategy is more Beneficial?

Why did some experts not select the strategy that could compensate for the timing variability? One possible explanation is that these subjects avoided a certain amount of risk. To achieve a longer TSZ, it is necessary to put the peak of the time-series vertical error curve in the success zone. However, it seems that using such a hand trajectory pattern increases the possibility of generating a “non-hit trajectory” (Figure 2D), compared to a pattern in which the peak is above the success zone (e.g., Experts 1 and 4 in Figure 4). Indeed, Expert 3, whose average TSZ was 9.0 ms (Figure 4), generated non-hit trajectories in 33% of all his throws, and his performance was the lowest in the expert group (performance error = 25.2 mm, success rate = 53.5%). Thus, such a pattern can compensate for the timing variability, but has an inherent risk of producing non-hit trajectories. Sternad et al. [24] also reported that in a virtual throwing task, some subjects selected strategies in the high penalty area, whereas others avoided risky strategies. It is assumed that the individual differences in selecting strategies that contain risk reflect the preferences of the individuals [25]–[27]. In the current study, the strategy selected by a thrower may have reflected their individual attitude toward the risk of producing “non-hit trajectories”.

The curves of the time-series error for the novices were more variable than those of the expert group. However, this variability did not simply indicate the “noise” of trial-to-trial variability. The variability in the early phase of motor learning indicates the exploration of the task and aids in finding the best solutions to achieve the desired result [28]. Almost all of the successful throws by novices involved the pattern that avoided the risk of producing a non-hit trajectory (red lines for novices, shown in Figure 4). However, a few throws had longer TSZs and produced successful hits (Novice 2 in Figure 4). If this pattern repeatedly compensated for the timing error and resulted in success, the thrower would learn this beneficial pattern. Through exploring the best solution, the throwers who sufficiently experienced a complementary pattern could begin to adopt such a strategy. On the other hand, the throwers who had insufficiently experienced such a pattern may have reduced their timing variability. Alternatively, throwers who can reduce the release timing variability to achieve the desired performance may not need to learn a complementary hand trajectory pattern.

In the current study, our two best throwers used different strategies. Therefore, we cannot conclude that one strategy was more beneficial than the other for consistently throwing accurately. Throwing darts is performed in a stable situation and is not affected by the environment or other players. Moreover, it is a relatively simple motion that is mainly conducted using elbow flexion and extension. These characteristics may enable throwers to better control their release timing by ensuring low variability. If a task involves a skill performed from an unstable position, or in an environment that is continuously changing, such as a jump shot in basketball or a fielder throwing a ball in baseball, the complementary strategy would be more beneficial for producing accurate and precise throws.

In general, the most optimal way to improve throwing accuracy and precision is to reduce movement and timing variability. The results of the current study indicate that shaping the hand trajectory to follow a complementary pattern may be as effective as reducing variability. Learners and coaches that understand this concept could utilize it in improving accuracy. Moreover, it should also be noted that the most beneficial strategy may change in response to the characteristics of the task and the individual.
